# Galactooligosaccharides Based on β-Galactosidase-Catalyzed Synthesis: Function, Biosynthesis and Optimization Strategy

**DOI:** 10.3390/foods15101803

**Published:** 2026-05-19

**Authors:** Bingyi Tao, Yiping Chen, Ren He, Tingting Huang, Shaoxiong Liang, Hongkun Chen, Xiaoping Rao, Xuchong Tang, Jianchun Jiang

**Affiliations:** 1 College of Chemical Engineering, Huaqiao University, Xiamen 361021, China; 2 Academy of Advanced Carbon Conversion Technology, Fujian Provincial Key Laboratory of Biomass Low-Carbon Conversion, Huaqiao University, Xiamen 361021, China; 3 BaYeCao Health Industry Research Institute Co., Ltd., Xiamen 361021, China; 4 Institute of Chemical Industry of Forest Products, Chinese Academy of Forestry, Nanjing 210042, China

**Keywords:** galactooligosaccharides, β-galactosidase, biosynthesis, prebiotics

## Abstract

Galactooligosaccharides (GOS) are one of the internationally recognized prebiotic products, which have become a hot research focus in the field of biofoods because of their strong prebiotic, sugar substitution and inflammation alleviation functions. β-galactosidase (Bgal) of different microorganisms is utilized industrially in order to achieve the biosynthesis of GOS. Although the biosynthesis of GOS has been supported by certain technologies, there is still room for further improvement of its synthetic yield. This paper mainly introduces the function and biosynthesis of GOS and its research progress in recent years to enhance the yield of biosynthesis. This paper also combines the research progress in related fields in recent years, based on the basic theories of molecular biology and bioinformatics, discusses the research progress of green, innovative approaches including enzyme engineering, enzyme immobilization, surface display, and microbial fermentation on the synthesis of GOS.

## 1. Introduction

Prebiotics, such as fructooligosaccharides, inulin, mannanoligosaccharides, xylooligosaccharides, and galactooligosaccharides (GOS), are non-digestible dietary components that can selectively stimulate the growth of probiotics without promoting the growth of potentially pathogenic bacteria, thereby providing health benefits to the host. As a result, they have become a research focus in the food industry and medical health [1,2,3]. GOS is one of the three major internationally recognized prebiotics. It is a non-digestible oligosaccharide found in small quantities in animal milk and exhibits functions including promoting gut health, serving as a sugar substitute, and exhibiting anti-inflammatory ability, and it is a commercially valuable functional food ingredient [4,5,6]. The use of GOS can be traced back to 1978 when Yazawa proposed replacing human milk oligosaccharides (HMOs) with GOS in powdered milk formulas to promote the growth of healthy intestinal probiotics in infants [7,8]. Soon after, the first commercialized product of GOS presented in Japan, and then the industrial production of GOS was put into practice all over the world, continuing to dominate the international market [9,10]. GOS has gained great attention from the dairy industry mainly due to the following reasons: (a) Lactose, which is inexpensive, is the main substrate for the synthesis of GOS and a major by-product in the dairy industry, resulting in a low synthesis cost of GOS [11,12]; (b) The biosynthesis of GOS can be completed by β-galactosidase (Bgal) through a two-step reaction, which is simple and well-established. The reaction features mild conditions and high conversion efficiency, and it is a reliable production method [6]; (c) GOS has a molecular structure and prebiotic function similar to HMOs, it has an advantage over other prebiotics when used as a preferred alternative in infant formula [5,13]; (d) GOS has direct or indirect medical functions, and the addition of GOS to dairy products can make them more healthful food products [14,15]. In addition, some people lose the ability to express *LacZ* either congenitally or acquiredly, without the assistance of microorganisms, they cannot efficiently degrade lactose in dairy products, thus suffering from lactose intolerance, which leads to problems such as flatulence and abdominal pain [11,16,17]. *Bifidobacterium longum*, one of the eight major probiotics in the human body, has a strong ability to encode Bgal and also has a high affinity for GOS [18]. Therefore, GOS can selectively promote the reconstruction of the intestinal microbiota, play the role of prebiotics, and ultimately produce several direct and indirect functions [19,20,21].

**Figure 1:**
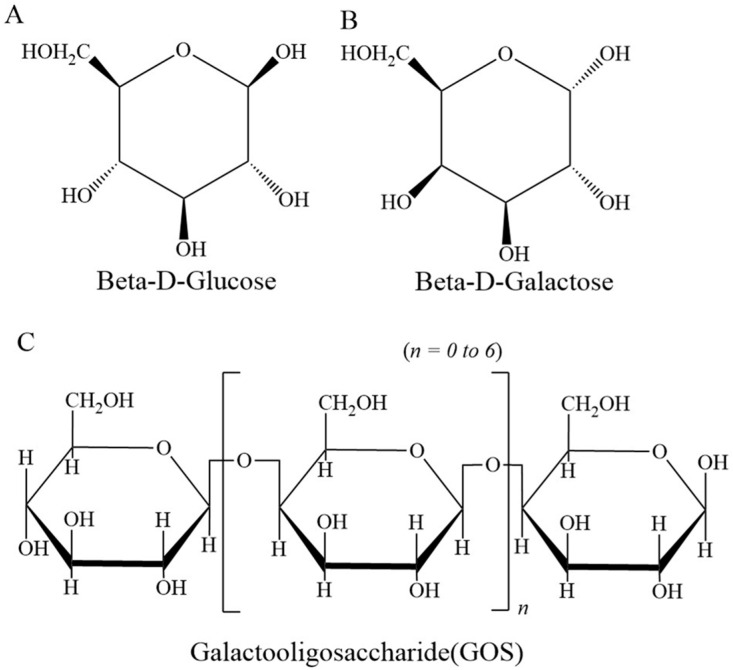
The structure of (**A**) β-D-glucose, (**B**) β-D-galactose, and (**C**) GOS.

GOS are oligosaccharides composed of 2–8 monosaccharide units and exhibit a variety of biological activities. There are mainly three production methods for GOS including natural extraction, chemical synthesis, and enzymatic production [22]. Among them, the yield and purity of the natural extraction method is relatively low. The GOS synthesis via chemical method is associated inevitably with issues such as toxic residues, low yields, and environmental pollution. Therefore, the enzymatic production method using Bgal to catalyze lactose is commonly employed in the industry [23]. This method boasts the advantages of high efficiency, low cost, mild conditions, and environmental friendliness. GOS are formed by using free galactose and glucose as substrates and linking them via β-glycosidic bonds [6,24,25]. The formation of β-glycosidic bonds is related to hemiacetal hydroxyls and free hydroxyls. Glucose and galactose possess hemiacetal hydroxyls and hydroxyls, and polymerization between hemiacetal hydroxyls and free hydroxyls can occur to generate glycosidic bonds, leading to the formation of oligosaccharides (Figure 1) [26,27,28,29]. The hydroxyl groups on galactose can polymerize with the hemiacetal hydroxyl group to form β-1,3-glycosidic bonds, β-1,4-glycosidic bonds, and β-1,6-glycosidic bonds to form GOS, and the formation and hydrolysis of these β-glycosidic bonds are controlled by the *LacZ* gene encoding Bgal [16,26,27,30]. Bgal is a glycosidase that was first discovered to exist in *Escherichia coli* by Beckwith in 1967 and has also been widely used in the relative study of gene expression regulation due to its ability to specifically degrade the β-glycosidic bond of X-gal [16,31]. Despite the convenient enzymes and biosynthetic pathways available for GOS, there are still certain production-related challenges, such as problems of purification and yield bottleneck [9,10]. Among these issues, GOS yield has been the focus of attention. Researchers have embarked on some innovative and effective methods are used to efficiently synthesize GOS to address these obstacles.

In recent years, the research on new types of Bgal and their industrial applications has been increasing steadily. However, most of the existing reviews still focused on the sources, functions, purification, characterization, catalytic parameters (such as temperature, pH and, lactose concentration), and kinetics of enzymes [32,33,34,35,36]. The core of these reviews is to enumerate the research on how to screen for enzymes featuring higher activity, greater stability, and a higher GOS yield. In contrast, the research reviews on discovering new applications or new technologies are still limited, especially regarding the discussions on the surface display technology and the microbial fermentation technology, which are two highly efficient production strategies, are still insufficient. Therefore, in order to fill this research gap, this review systematically explores the effects and potential mechanisms of technologies such as enzyme engineering, enzyme immobilization, surface display, and microbial fermentation on the synthesis of GOS. Meanwhile, this review provides a systematic summarize of the recognized prebiotic functions of GOS, and presents keyword co-occurrence cluster analysis for GOS and Bgal research. Finally, this article will provide an outlook on the future research directions and development trends of GOS synthesis based on the current technical limitations, with the aim of offering valuable insights for the continuous innovation in this field.

## 2. GOS and Its Functions

### 2.1. As a Prebiotic

**Figure 2:**
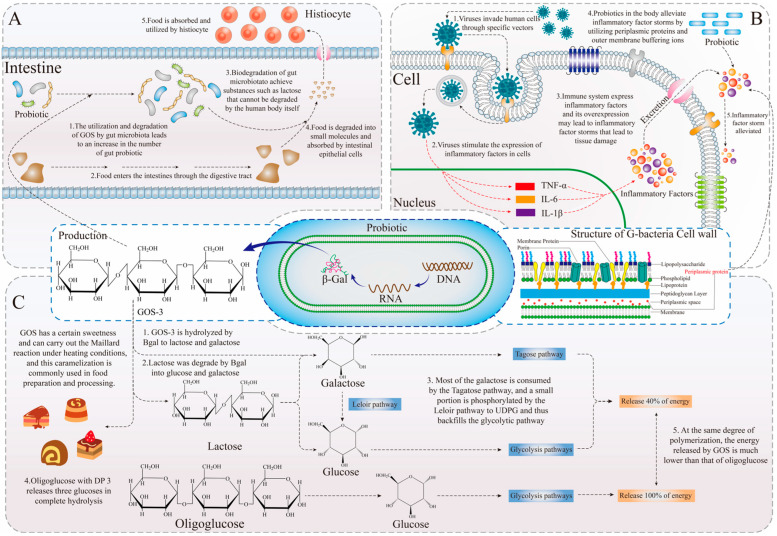
(**A**) GOS improves the stability of gut microbiota and promotes human digestion. (**B**) GOS as a prebiotic promotes the growth of probiotics and indirectly alleviates the inflammatory reaction in the body. (**C**) GOS as a sugar substitute produces less metabolic energy.

Lactose, a disaccharide formed by the polymerization of glucose and galactose through a β-glycosidic bond, is a common carbohydrate found in dairy products. Its degradation and metabolism in the human body are facilitated by Bgal [37,38]. Due to the lack of the *LacZ* gene in most individuals, which encodes the Bgal protein, lactose can accumulate in the body, leading to intestinal issues such as flatulence and diarrhea [17,39]. During digestion, GOS can selectively reach the intestine and stimulate the proliferation of beneficial intestinal flora that produce Bgal, thereby aiding in the degradation of accumulated lactose and improving intestinal health [14,25] (Figure 2A). Slavin et al. [40] noted that GOS enhances the digestive environment in animals by improving the composition of intestinal flora. Torres et al. [41] comprehensively described the physicochemical properties, physiological roles, and applications of GOS as a prebiotic for enhancing digestion. As a prebiotic product, GOS is currently the most widely used functional food ingredient, as it increases the abundance of beneficial intestinal microorganisms to improve digestion.

### 2.2. As a Sugar Substitute

When GOS are completely degraded by intestinal flora, free galactose and glucose are produced [42]. The degradation of GOS yields less glucose compared to the breakdown of polysaccharides in general. Additionally, some of the galactose generated during this process enters the tagatose pathway, indicating a reduced capacity for aerobic metabolism following galactose phosphorylation, which suggests a decrease in energy output [43,44,45] (Figure 2C). Under identical conditions, the complete degradation of GOS releases less energy than traditional oligosaccharides, and the resulting sweetness is approximately 20–40% that of sucrose [14]. Furthermore, due to its reaction with proteins during heating, GOS has found widespread application in the production of baked goods [15]. Additionally, research has demonstrated that GOS can promote weight loss by inhibiting the hypertrophy and hyperplasia of white adipose tissue while significantly increasing serum levels of total cholesterol, triglycerides, high-density lipoprotein cholesterol, and low-density lipoprotein cholesterol [46]. For patients with obesity and fatty liver, GOS serves as an effective sugar substitute that can enhance dietary quality, indicating a promising future for GOS in the field of sugar alternatives [47].

### 2.3. As an Indirect Anti-Inflammatory Agent

In a symbiotic system, two interspecies in a symbiotic relationship survive in the same environment, and they will simultaneously maintain the environment in which they live for the benefit of each other [48]. Similarly, when people is attacked by a pathogen and generates an immune response, numerous immune cells in the vicinity will respond rapidly by producing large amounts of inflammatory factors such as IL-6, IL-β, TNF-α, etc. [49,50]. Excessive accumulation of these inflammatory factors will cause corresponding tissue inflammatory responses, leading to elevated body temperatures, which will in turn lead to metabolic disorders and tissue damage [51,52]. Therefore, in order to maintain the environmental homeostasis of the symbiotic system, the intestinal flora will use the buffering components in their cell walls to achieve the mitigation of inflammatory responses in their periphery, thus playing an important regulatory role in the regulation of the body’s homeostasis of the internal environment [20] (Figure 2B). Wu et al. [53] found that GOS and *Limosilactobacillus reuteri* synergistically alleviate gut inflammation and barrier dysfunction. Tang et al. [49] transplanted the fecal bacteria to achieve the alleviating therapeutic effect of using probiotics on acute lung injury in mouse lungs induced by lipopolysaccharide. Meanwhile, Wang et al. [51] utilized GOS to have a significant alleviating effect in mouse cells undergoing intestinal barrier damage and inflammatory response. It is evident that the proliferative effect of GOS on the intestinal flora will indirectly alleviate the inflammatory response. In addition, Arnold et al. [1] stated that GOS achieves modulation of intestinal flora to alleviate inflammation by enhancing the composition of the intestinal flora and regulating changes in gene expression of inflammatory factors in people. On the one hand, in terms of gut microbiome, the use of GOS will increase the abundance of Bgal, which in turn increases the abundance of glycolytic bacteria. On the other hand, in terms of regulation of animal genome expression, the expression level of the inflammatory factor TNF-α is reduced in animals after GOS treatment [49,51,52,54]. In the medical field, GOS as a dietary supplement has been shown to have great effects in inflammation treatment, and the application of GOS in inflammation treatment will be further investigated in the future.

## 3. Biosynthesis of GOS

**Figure 3:**
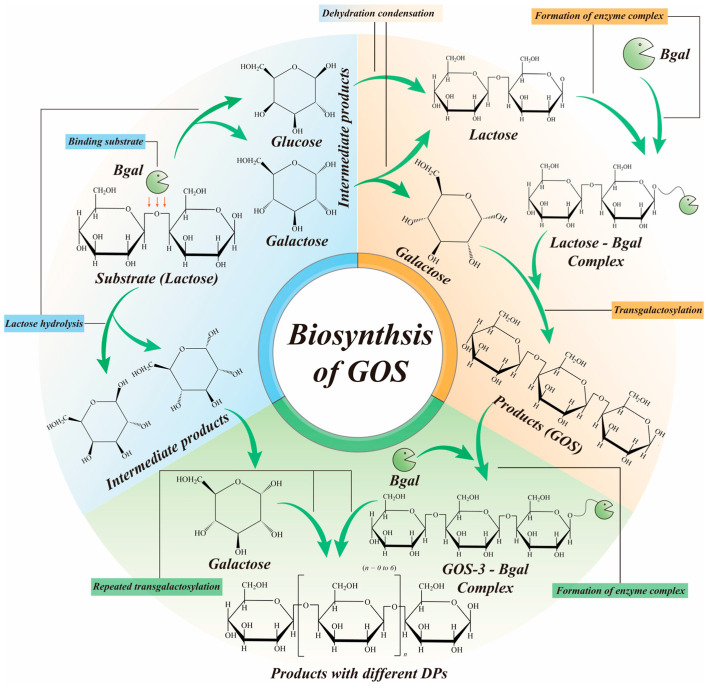
The biosynthetic pathway of GOS completed by Bgal.

The primary synthesis of GOS involves the hydrolysis of lactose and successive transgalactosylation reactions. Lactose and galactose are linked via β-glycosidic bonds to form GOS [19,55,56]. Transgalactosylation is a polymerization reaction that uses the hydroxyl group on galactose as a substrate and uses the hydroxyl group on galactose to link with the hemiacetal hydroxyl group of other monosaccharides [27]. The synthesis process of GOS catalyzed by Bgal involves the following steps (Figure 3) [26]. Lactose is a disaccharide formed from glucose and galactose through a β-glycosidic bond. During hydrolysis, the galactose on lactose binds to the active site of Bgal [30,57]. Bgal subsequently severs the glycosidic bond between galactose and glucose, forming a galactose-enzyme complex and releasing free glucose [58]. The formed complex binds to another lactose, and the Bgal in the complex catalyzes a transgalactosylation reaction between lactose and galactose, resulting in a β-glycosidic bond and the formation of GOS-3 [27,59]. After the formation of GOS-3, Bgal detaches from the complex in order to bind to the next molecule of galactose [32]. The detached enzyme will continue to bind the next galactose, forming a complex, which in turn binds to lactose or GOS-3, which undergoes a similar transgalactosylation reaction to form GOS-3 or GOS-4. In this way, the cycle is repeated to form higher-degree polymerization GOS [60]. Eventually, lactose is thoroughly hydrolyzed to glucose and galactose for the synthesis of different polymerization GOS.

### 3.1. Bgal

In the industrial production of healthy dairy products, Bgal is often used to obtain low-lactose dairy products in order to avoid the digestive problems associated with the accumulation of lactose in dairy products consumed by lactose intolerant patients [61,62,63]. In addition, the degradation products of lactose can also be utilized by Bgal to be converted into more commercially valuable oligosaccharides, including GOS, through a transgalactosylation reaction [64,65]. These properties endow Bgal with great production value in dairy industrial production, and the utilization of Bgal to achieve the removal of lactose and the addition of functional oligosaccharides in dairy products has become the main content of the current dairy industrial production [29,30,58].

Bgal is widely distributed in animals, plants and microorganisms [16,31]. Commonly employed microbial strains include *A. oryzae*, *K. lactis*, *E. coli*, *Bifidobacterium bifidum*, *Enterobacter cloacae*, *Streptococcus thermophilus*, *Lactobacillus delbrueckii*, *Lactobacillus plantarum*, *Pichia pastoris*, *Thermothielavioides terrestris*, *Thermotoga naphthophila*, *Pseudomonas tritici*, *Sulfolobus solfataricus* and *B. circulans*. The yield of these strains of Bgal for GOS production varied (Table 1). In addition, Bgal have active sites formed by the spatial folding and coiling of amino acids, which is responsible for catalyzing a variety of reactions [66]. Therefore, environmental factors will greatly affect the activity of Bgal and thus affect the GOS yield. The environmental factors that determine the GOS yield mainly include temperature, pH, reaction time, enzyme concentration and lactose concentration [9,64]. Temperature, pH, enzyme concentration and lactose concentration are important factors affecting the kinetic parameters of enzymatic reactions and have been used as the focus of optimization in biocatalytic reactions [19,24,25,67]. Firstly, temperature and pH significantly affect GOS synthesis catalyzed by Bgal. Bgal derived from *Kluyveromyces lactis* can obtain a GOS yield of 26% at a suitable temperature of 40 °C, while low temperature affects the state of intermolecular motility, which in turn affects the catalytic activity of the enzyme, so that the rate of GOS yield suffers, and the yield only 8.34% at 7 °C [58,68]. Bgal from *Aspergillus oryzae* had a GOS yield of 24.3% at an optimal pH of 6, and its GOS yield increased by 8.3% compared to the condition at pH 4.3 [69,70]. In addition, the effect of lactose concentration on GOS yield has been consistently demonstrated, with higher initial lactose concentrations resulting in increased GOS production [42]. Frenzel et al. [68] performed GOS biosynthesis at an initial substrate concentration of 40% in a study using Bgal from *Bacillus circulans*, ultimately converting 41% of the substrate to GOS, whereas Rodriguez-Colina et al. [71] converted only 16.5% of the substrate to GOS at an initial lactose concentration of 4.5%. During the reaction, an appropriate concentration of enzyme leads to a more rapid lactose hydrolysis and transgalactosylation reaction, which in turn improves the efficiency of GOS production. Singh et al. [42] achieved 12.63% GOS yield by controlling the Bgal concentration of *K. lactis* at 7.14 U/mL, whereas only 8.34% of GOS could be produced at 2.5 U/mL. It is worth noting that timely collection of GOS from the reaction system is one of the ways to improve the yield during GOS synthesis. This is due to the fact that the lactose hydrolysis reaction catalyzed by Bgal and the transgalactosylation reaction proceed simultaneously, too early collection of the reaction product makes the lactose hydrolysis incomplete, while too late recovery of the reaction product hydrolyzes the product, which in turn affects the yield [59]. Singh et al. [42] confirmed the optimal time of recovery of Bgal from *K. lactis* for the production of GOS in the production process of low-fat milk, and the recovery of Bgal at collection at 4 h gives maximum GOS synthesis yield of 12.63%, while recovery at 24 h gives only 4.96% GOS synthesis yield. Until now, the process conditions for GOS production have been examined by researchers, and optimization efforts are becoming well established. Frenzel et al. [68] explored the synthesis of GOS by Bgal from *A. oryzae* under reaction conditions of pH 4.5, incubation temperature of 40 °C and 40% initial lactose concentration, which resulted in the conversion of 21% of the lactose to GOS. Furthermore, Bgal from different sources can lead to different yields of GOS under the same reaction conditions. De Albuquerque et al. [58] reported that it possessed a 26% GOS yield under optimal production conditions such as pH 6.5 and 40 °C operating temperature, and *K. lactis* demonstrated a more efficient lactose utilization compared to *A. oryzae*. It is worth mentioning that *B. bifidum* and *B. longum* are the first intestinal flora stimulated by the prebiotic action of GOS occurrence, and their Bgal realized the conversion of 53.1% and 50% of lactose to GOS, respectively, under their respective optimal production conditions, and *B. bifidum* and *B. longum* showed great production value in GOS production [18,72]. Therefore, in industrial production, the use of enzymes is a key variable in determining the yield of GOS synthesis. In addition, Bgal from some extreme thermophilic bacteria possesses strong catalytic activity and high temperatures of reaction to avoid the contamination of the production process. Wu et al. [73] reported that Bgal from *S. solfataricus* at pH 6.5 and an operating temperature of 75 °C could obtain a GOS yield of 50%. Regrettably, despite the stronger performance of this type of Bgal in synthesizing GOS, there are few cases of industrial production of dairy products, which is mainly due to the food safety problems associated with the use of thermophilic Bgal in the production process [10]. Therefore, the microbial source of Bgal and its corresponding reaction environment common determine the synthesis efficiency of GOS, and biosynthesis of GOS focuses on optimizing the catalytic activity and stability of Bgal. Meanwhile, Bgal can be improved the performance through protein engineering and enzyme immobilization technology in the future. It is expected to unleash its high-efficiency catalytic potential while ensuring safety and stability, thus promoting the upgrading of functional dairy products.

### 3.2. Directed Mutation of Bgal

Industrially produced GOS are generally complex mixed systems that include different polymerization GOS [25]. Different polymerization GOS are formed by the catalysis of different sources of Bgal, which have different catalytic specificities [9]. The Bgal of some bacteria has very strong catalytic activity, but the convergent catalytic reaction may be detrimental to GOS synthesis. For example, the enzyme lineage of *B. circulans* is more inclined to GOS-3 synthesis, whereas the enzyme lineage of *K. lactis* is more inclined to hydrolyze lactose, which, despite possessing high catalytic activity, is unable to adequately perform the transgalactosylation reaction for GOS synthesis [27,42]. Some studies proceeded to solve the above problems, researchers used different sources of Bgal, combined with SWISS-Prot, Alphafold to analyze the peptides, to achieve the prediction of the protein structure, signal peptide sequence and distribution, protein physicochemical properties, and functional calibration of the polypeptide sequences after translation, in order to determine the active sites that need to be mutated [89,90,91,92]. This was followed by multiple sequence analysis using Clustal X, which was combined with the results of the analysis to show what needed to be modified to form the ideal Bgal catalytic activity for effective modification of the active site [89]. Liao et al. [93] engineered a Bgal^L764T/V842G^ using multistrategy engineering including sequence alignment, flexible regions modification, and model prediction, its GOS yield is 36% higher than that of the wild type. The GOS yield of the Bgal combinatorial mutant derived from *A. oryzae* obtained through semirational design is 1.82 times that of the wild type [94]. Yu et al. [95] employed three structure-based strategies as well as machine learning MECE platform to screen for the optimal Bgal mutant. Under the condition of using 35% (*w*/*v*) lactose as the substrate, the GOS yield of the H331V mutant reached 76.57%. Meanwhile, researchers will also provide Bgal genes that can be utilized in Genbank for subsequent further optimization studies [96]. *S. thermophilus* is an extremely thermophilic bacterium whose Bgal can efficiently undergo lactose hydrolysis but is unable to pass sufficiently through transgalactosylation to produce the reaction substrate. Therefore, Zhao et al. [62] used the Swiss-Model protein modeling server to analyze the three-dimensional structure of Bgal and found the active sites Tyr801 and Pro802. Clustal X was used to analyze the sequence difference in Bgal in multiple strains of wild bacteria, and site-specific mutation of Tyr801 and Pro802 into His801 and Gly802 was achieved to achieve directional modification of the active site of transgalactosylation. The mutated Bgal of *S. thermophilus* has stronger ability to undergo the transgalactosylation reaction, which, when used for GOS production, boosted the GOS yield by nearly 224% compared to the wild-type strain [62]. In addition, Wu et al. [73] achieved an increase in the synthesis yield of GOS by introducing cysteine into the catalytically active globular protein subunit in Bgal of *S. solfataricus*, which resulted in the formation of a disulfide bond and improved the stability of the protein subunit, and the final liquid achieved an increase in the synthesis yield of GOS. In short, the precise modification of the active site of Bgal can alter the enzyme’s activity, thereby increasing the yield of GOS. This implies that the design of high performance Bgal has become a major research focus in the study of GOS synthesis.

### 3.3. Immobilization of Bgal

**Figure 4:**
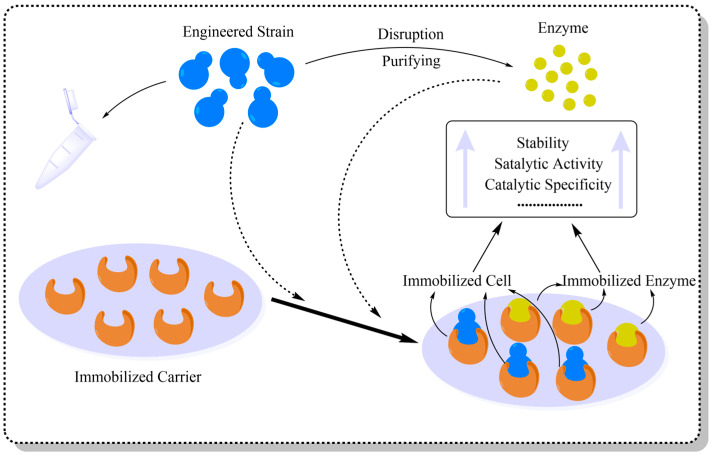
Preparation of immobilized cells and enzymes using biological materials.

In traditional enzymatic synthesis of GOS, Bgal is typically used in a free form. However, this catalytic system has limited stability and Bgal is susceptible to interference from environmental factors, which may result in high production costs [97]. Since the rapid development of materials science, immobilization has become a center of intersection between materials and biological disciplines [98]. The materials used for immobilization have the advantages of inexpensive raw materials, stability and good biocompatibility [30]. During the culture process, immobilized materials adsorb and immobilize free bacteria and Bgal in the culture system, thus restricting the mobility of proteins or cells and immobilizing them without interfering with their functions [99]. When an enzyme or cell is immobilized on an inert carrier, it will not only allow substrate exchange and product efflux, but at the same time it will limit the changes in the enzyme conformation due to changes in pH or temperature, thus reducing the sensitivity of the natural enzyme and the cell to these physical parameters, and thus increasing the catalytic stability and efficiency of the enzyme (Figure 4) [81]. These properties allow bacteria to take into account the advantages of immobilized materials and the catalytic properties of the enzyme during metabolism, which leads to the improvement of the catalytic activity and stability of the enzyme and an increase in the yield of the GOS. Currently, the widely used immobilization materials and their effects are organized as shown in Table 2. Among them, magnetic nanocarriers of chitosan and metal oxides are widely used for the immobilization of Bgal because of their good biocompatibility and recycling properties, as well as their combined advantages of improving enzyme stability and activity. Alnadari et al. [67] prepared a chitosan-magnetic nanocarrier and used the carrier for the immobilization of Bgal. Compared with the fermentation of free Bgal, the catalytic activity of immobilized Bgal was up-regulated by a factor of 1.4 at 55 °C, the enzyme activity was up-regulated by a factor of 1.6 at an optimal pH of 6.6, and the GOS yield was up-regulated from 24% to 28% after immobilization. Urrutia et al. [56] immobilized Bgal on amino, carboxyl, and chelated ethylenediacyl agar, and the amount of contact protein of immobilized Bgal was significantly higher than the free Bgal. In addition, Córdova et al. [75] develop a magnetic responsive biocatalytic membrane reactor employing immobilized *A. oryzae* Bgal on iron oxide super-paramagnetic beads. Although enzyme immobilization reduced the GOS yield from 25% to 10%, it effectively doubled the specific productivity and flux. It can be seen that immobilization has become one of the current strategies for the optimization of GOS production. The immobilized use of Bgal and papain has also been documented in the application of natural carrier agarose, and the good biocompatibility and material properties of sodium alginate and chitosan have been reported in the maintenance of lipase and protease stability [99,100]. Using the glutaraldehyde cross-linking method, Bgal is attached to the anion-exchange resin to form an immobilized biocatalyst, which significantly regulated the balance between transgalactosylation and hydrolysis activities, while simultaneously enhancing the yield of GOS and the stability of Bgal [101]. Xuan et al. [102] has developed a novel carrier-free cell immobilization method, which utilizes genipin to cross-link *K. lactis* CGMCC 2.1494 capable of producing Bgal, thereby exhibiting higher thermal tolerance and organic solvent tolerance. In summary, the immobilization strategy significantly enhances the catalytic stability, yield, and flexibility of Bgal. Moreover, different carriers and cross-linking methods have varying effects on the activity and stability of Bgal. Therefore, immobilization does not simply aim for maximum yield but should seek a balance among stability, recyclability, catalytic efficiency, and product yield. In the future, designing suitable immobilization materials for specific Bgal sources and reaction environments will be the key to achieving efficient, stable, and recyclable production of GOS.

### 3.4. Surface Display

Although the effective reuse of enzymes over multiple reaction cycles has been achieved through immobilization, factors such as the high cost of immobilization materials, the complexity of the immobilization procedure, the loss of enzyme activity, and the difficulty in purifying the enzyme and products have limited the immobilization of traditional enzymes [109]. In recent years, surface display has been recognized as a green means of gene editing that can efficiently improve the catalytic activity of enzymes in fermentation systems, and cell wall anchoring proteins, represented by *Saccharomyces cerevisiae* α-agglutinin, have been widely used in the fusion formation of various proteins [110]. The surface display technology entails linking the target protein to the anchoring protein and subsequently introducing it into the host cell for expression. The anchoring protein is expressed, the target protein is also expressed and localized on the surface of the host cell, thus achieving the surface display of the target protein [111]. Subject to the action of anchoring proteins, the recombinant fusion proteins are usually enriched on the cell surface, thus improving the catalytic efficiency and catalytic stability of the enzyme [112]. Table 3 summarizes the research on surface display in recent years. The autotransporter of esterase Est7 guaranteed a surface display ratio of 89.67% for Est7 in *E. coli*. The displayed Bgal retained 41.41% activity in sixth batch, indicating the considerable potential of E7AT in developing efficient whole cell catalysts [113]. Researchers employed surface display technology to present the Bgal derived from *A. oryzae* on the surface of *Yarrowia lipolytica*. When this engineered strain utilized high-concentration lactose as the substrate at 60 °C, it produced 160 g/L of GOS and could be reused at least 10 times. Moreover, its thermal stability and reaction efficiency were significantly superior to those of free enzymes [114]. Researchers constructed the formation of surface displayed biofilms by knocking out *PAS_chr1-3_0226* gene in *P. pastoris* and overexpressing the *Pir1p* gene from *S. cerevisiae* S288c and the *LacA* gene from *A. oryzae* respectively. These biofilms were attached to cotton fiber materials for immobilized fermentation, and the continuous catalytic batches could be repeated up to 23 times, and the harvested GOS yield was 50.3% in 500 g/L lactose solution [115]. The study found that through a systematic investigation of 10 genes associated with the biofilm formation of *K. phaffii*, it has been discovered that the KpFlo11C domain of BSC1p promotes the aggregation of biofilm cells on the carrier. This domain was overexpressed in the *K. phaffii* cell display system to obtain enzyme-cell@material biocatalyst, which continuously and robustly produced GOS at a rate of 8.16 g/L/h in a 6 L fermenter [116]. In addition, Bgal from different sources exhibit distinct performance characteristics. *Lactobacillus delbrueckii* subsp. *bulgaricus* has been proven to outperform *Lactobacillus reuteri* in terms of both immobilization yield and the amount of active surface-anchored enzyme [117]. However, the application of surface display still has certain limitations, due to the limitation of anchor protein sources, and the number of model strains that can be used for surface display modification is still very limited [118]. Moreover, the pressure of the fermentation environment and factors related to cell autolysis may lead to a decrease in the enzyme’s activity and catalytic efficiency [119]. Meanwhile, the display is still restricted by the low efficiency of heterologous proteins and poor display efficiency [120]. Therefore, in the future, the key to promoting the industrialization of surface display in GOS production lies in the exploration of novel anchoring protein elements, the optimization of the secretion and folding pathways of host cells, and the construction of an efficient and stable surface display platform through the integration of synthetic biology strategies.

### 3.5. Microbial Fermentation for GOS Production

The production of GOS in the dairy industry often uses Bgal from *A. oryzae*, *K. lactis*, *B. longum* as a catalytic enzyme, although these enzymes have the advantages of easy accessibility and high productivity, the disadvantages of a short catalytic cycle and a small production system still need to be urgently solved [10,24,30,42,69]. Microbial fermentation is considered as a solution that can be used to solve the above problems [121]. However, the fermentation process reduces the reaction substrate for transgalactosylation due to the depletion of substrates for GOS synthesis by non-essential metabolic pathways, resulting in a generally lower yield of GOS synthesized by microbial fermentation than enzyme-catalyzed synthesis, which ultimately affects the GOS yield [37,43,45]. Therefore, some studies have proceeded to further molecular modification and regulation of Bgal to improve the GOS yield by efficiently utilizing protein tags and thus constructing an efficient and stable fermentation catalytic system.

**Figure 5:**
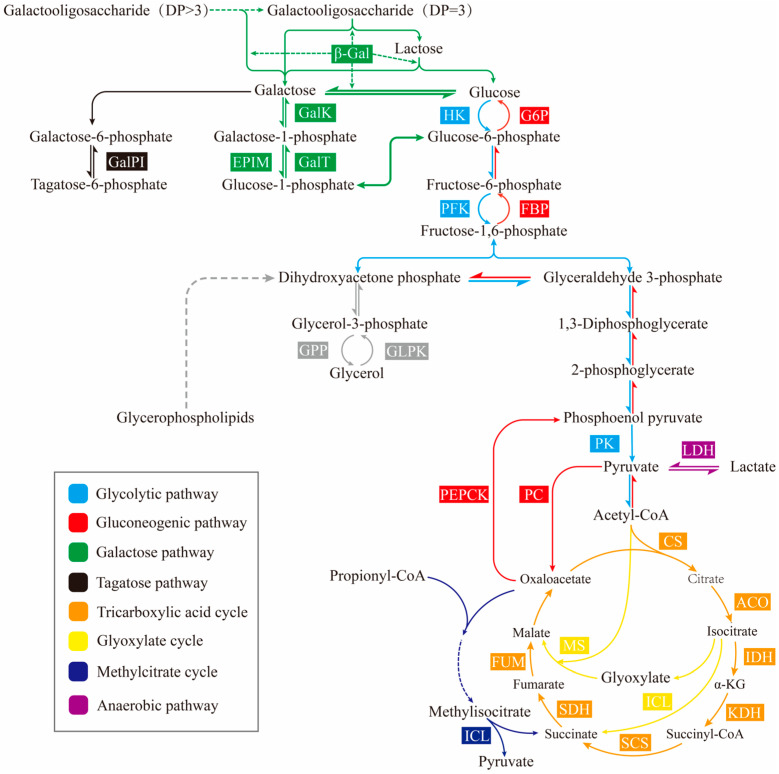
Main metabolic consumption pathways of GOS. DP Degree of polymerization, HK Hexokinase, PFK Phosphofructokinase, PK Pyruvate kinase, G6P Glucose-6-phosphatase, FBP Fructose-1,6-bisphosphatase, PEPCK Phosphoenolpyruvate carboxykinase, PC Pyruvate carboxylase, β-Gal β-Galactosidase, GalK Galactose kinase, GalT Galactose-1-phosphate uridyltransferase, EPIM UDP-galactose-4-epimerase, GalPI Galactose-6-phosphate isomerase, GPP Glycerol-3-phosphate phosphatase, GLPK Glycerol kinase, LDH Lactate dehydrogenase, CS Citrate synthase, ACO Aconitase, IDH Isocitrate dehydrogenase, KDH α-Ketoglutarate dehydrogenase, SCS Succinyl-CoA synthetase, SDH Succinate dehydrogenase, FUM Fumarase, ICL Isocitrate lyase, MS Malate synthase, ICL Methylisocitrate lyase, α-KG α-Ketoglutarate.

Currently, the metabolic pathways present in microbial fermentation of GOS and its synthesized substrates are shown in Figure 5. Represented by lactose as a substrate, the decomposition products of lactose are glucose and galactose [42,84]. Among them, glucose is mainly consumed by the glycolytic pathway to provide the necessary energy for growth and development, while galactose as a substrate mainly occurs in the transgalactosylation reaction and plays a key role in the synthesis of GOS [12,45]. During fermentation, galactose is mainly consumed by the tagatose pathway and the Leloir pathway. The tagatose is produced by L-arabinose isomerase catalyzing the isomerization of galactose, whereas microorganisms with the ability to express L-arabinose isomerase also have the ability to express Bgal, which affects the GOS production rate by competing with Bgal for the binding of galactose [45]. Therefore, the effect of L-arabinose isomerase on GOS synthesis yield deserves to be further considered when utilizing these microorganisms for fermentation. On the other hand, the leloir pathway, which is widely distributed in dairy-producing strains, becomes a non-essential metabolic depletion pathway that hinders GOS synthesis by converting galactose to glucose-6-phosphate and thus entering the EMP pathway through a multistep catalytic reaction of galactose kinase (GALK), galactose-1-phosphate uridyltransferase (GALT), and UDP galactose-4-differential isomerase (UDPG) [43]. Ponnusamy et al. [122] further increased the GOS yield by removing the key enzymes of the leloir pathway, GALK, GALT and UDPG, in *K. lactis*, thus circumventing the consumption of free galactose by the leloir pathway in the bacterium, and ultimately achieving a yield increase of 349% [122]. During fermentation, the consumption of galactose became a key factor affecting the synthesized GOS, and the non-essential metabolic consumption pathway affected the synthesized GOS yield. Gene editing has now solved many production and purification challenges through the regulation of expression levels of key sequences, the efficient use of protein tags, and the knockout of key sequences in non-essential metabolic pathways [123]. Zhao et al. [124] generated the engineered strain *Bacillus licheniformis* H107-06A via metabolic engineering, specifically through remodeling of the central carbon metabolism pathway and knockout of GOS metabolism. Under anaerobic conditions, strain H107-06A co-produced 141.89 g/L of GOS from syrup. In summary, microbial fermentation offers a viable approach to overcome the limitations of traditional enzyme catalysis including short catalytic cycles and small reaction systems. However, GOS yield is often restricted by the competitive consumption of galactose through non-essential metabolic pathways. Therefore, rationally designing microbial cell factories to block the by-pass consumption of galactose and enhance its flux towards transgalactosylation is the efficient strategy for improving the efficiency of GOS synthesis via fermentation. In the future, by metabolic engineering and high throughput screening techniques to further optimize the metabolic network of host strains, it is expected to achieve efficient, green, and large-scale fermentation production of GOS.

### 3.6. Keyword Co-Occurrence Cluster Analysis

**Figure 6:**
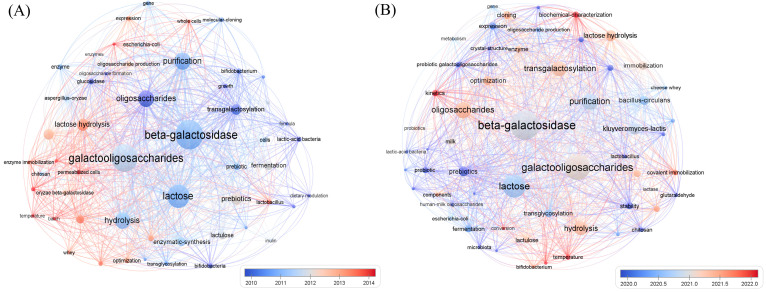
Co-occurrence networks of all keywords related to β-galactosidase and galactooligosaccharides. Timeline visualization of co-occurring keywords in the galactooligosaccharides production field from 1999 to 2016 (**A**) and 2017 to 2026 (**B**) respectively. The node size indicates keyword frequency, and the color represents the average publication year (blue = older, red = more recent).

VOSviewer 1.6.20 software was employed to perform keyword co-occurrence cluster analysis on galactooligosaccharides and β-galactosidase for two periods, 1999–2016 and 2017–2026, with a keyword frequency threshold of ≥5 (Figure 6). The analysis revealed that the core research objective remained consistent across both periods, with the synthesis of GOS from lactose catalyzed by Bgal and the reaction mechanism consistently centered on lactose hydrolysis and transgalactosylation. Additionally, fundamental research areas, including enzyme purification, process optimization, prebiotic applications, and traditional microbial sources of Bgal, received sustained attention throughout the entire period. Lactose was the primary substrate for GOS synthesis, subsequently, research focus gradually shifted toward the utilization of whey, a by-product of the dairy industry. The application of GOS was predominantly concentrated in the fields of functional foods and prebiotics.

From 1999 to 2016, research focus transitioned from basic enzymology to industrial application technologies, with enzyme immobilization emerging as a research hotspot. For instance, immobilization of Bgal using natural carriers such as chitosan was reported in previous studies [108]. Increased attention was also paid to optimizing reaction conditions (e.g., temperature, pH, and substrate concentration) for enzymatic GOS synthesis, as well as the efficient utilization of low-cost substrates such as whey [88]. Concurrently, more Bgal enzymes from diverse microbial sources were discovered, such as *Lactobacillus* spp. [125] and *B. circulans* [56], and *K. lactis* [126]. From 2017 to 2026, research hotspots continued to focus on the optimization of enzyme immobilization technology and the exploration of novel Bgal sources. Notably, enzyme immobilization technology has advanced from simple physical adsorption to more stable methods such as covalent cross-linking. In addition, novel carriers including magnetic nanoparticles and metal–organic frameworks have been widely adopted to enhance enzyme stability and reusability, thereby reducing the cost of industrial GOS production [30,33]. In recent years, with the rapid development of structural biology, synthetic biology, and machine learning technologies, research on Bgal molecular modification and GOS synthesis has entered a stage of precise design and functional customization. Yu et al. [95] applied the MECE principle to efficiently predict beneficial mutation sites of Bgal, accurately identifying distal mutation sites that are difficult to detect via traditional rational design. Meanwhile, enzyme kinetics has been deeply integrated with enzyme crystal structure analysis, biochemical characterization, and molecular modification techniques. By comparing changes in enzyme kinetic parameters and structural characteristics before and after mutation, the intrinsic regulatory mechanisms of the enzyme active center and its microenvironment on catalytic efficiency and transglycosylation properties have been further elucidated, facilitating in-depth exploration of the Bgal catalytic mechanism [93,103,^127^,,^128^]. Collectively, over the 27-year study period, research in this field has been consistently focused on low-cost and high-efficiency GOS synthesis using Bgal, evolving sequentially from fundamental enzymology and enzyme source screening, through catalytic process optimization, to the current stage of precise molecular design and in-depth catalytic mechanism elucidation enabled by cross-disciplinary technologies.

## 4. Conclusions and Future Trends

In the dairy industry, GOS is an ideal alternative to HMOs due to its strong targeting and prebiotic effect. Bgal is an important tool enzyme catalyzing the synthesis reaction of GOS, and its catalytic activity and reaction conditions remain the key factors influencing GOS yield. Despite the isolation of numerous Bgal with diverse properties from a wide range of microorganisms, these enzymes still face numerous limitations such as poor stability, low reusability, and limited substrate tolerance in actual industrial production. The technology of enzyme immobilization and enzyme engineering have been exploited to modify Bgal for better catalytic properties and stability. Enzyme immobilization technology has advanced significantly; however, developing a universal material compatible with Bgal from diverse sources remains a challenge owing to the structural diversity, differences in molecular weight, and the selective binding preferences for specific carrier materials of enzymes. For further development, the engineering of Bgal is guided by emerging computational approaches including de novo design, machine learning and artificial intelligence with predictive and precise accuracy by analyzing protein features and functions. While enzymatic GOS synthesis has been greatly advanced by both established and emerging technologies, it lacks the simplicity and inexpensive nature of quantitative production by microbial fermentation. Therefore, we still need to seriously consider the advantages of microbial fermentation for GOS production. Based on the metabolic engineering canonical approach, further studies can explore the synthesis of GOS using an integrated approach of metabolomics, proteomics and bioinformatics. In this case, reducing the consumption of non-essential metabolic pathways in fermentation strains and surface display are direct solution. It is worth noting that surface display and its dependence on the formation of biological membrane wall system, the enrichment of membrane wall system formation can enable the fusion proteins to efficiently bind to the cell surface and form more surface proteins, thus preventing the substrate to be transferred into the membrane to be consumed by non-essential metabolic pathways, in order to maximize the transgalactosylation reaction and obtain the desired GOS yield. In addition, there are other pressing challenges for the synthesis of GOS. As a complex hybrid system, the reaction mixture is a mixture of monosaccharides and GOS with different degrees of polymerization, and the separation and purification of these substances is also a challenging task. Overall, significant challenges persist in the industrial-scale production of GOS, and further interdisciplinary research is required to address these issues.

## Figures and Tables

**Table 1:** Statistics on the performance of different sources of Bgal.

Source of Enzyme	Lactose Concentration (%)	pH	Temperature (°C)	Yield (%)	References
*A. oryzae*	19	6.0	30	24.3	[69]
	40	4.5	40	21.0	[68]
	40	6.5	60	13.0	[68]
	40	4.5	50	59.9	[74]
	47	4.5	55	25	[75]
*B. bifidum*	40	6.0	40	27.0	[76]
	40	6.5	45	44.2	[24]
*B. circulans*	50	5.08	30	50.6	[27]
	40	7.0	40	41.0	[68]
	4.6	6.7	55	43	[77]
	6	6.5	10	82	[78]
*K. lactis*	16	6.7	7	12.6	[42]
	40	6	40	–	[79]
	23	6.5	40	26.0	[58]
	15	7.2	35	21.7	[80]
*L. plantarum* CICC22186	40	7.0	35	30.0	[64]
*S. thermophilus*	5	6.5	42	20.5	[62]
*S. thermophilus* BgaQ8012	5	6.5	42	26.7	[62]
*T. terrestris*	13.7	4.0	60	19.4	[65]
*S. solfataricus*	60	6.5	75	50.0	[73]
*E. coli*	1	6.5	60	5.6	[81]
*E. cloacae*	38	7	40	67	[82]
*L. delbrueckii* subsp. *bulgaricus* 43	20	6.5	55	34.0	[83]
*L. delbrueckii* subsp. *bulgaricus* CRL450	30	6.5	45	41.3	[84]
*P. pastoris*	50	6.0	50	50.3	[85]
*T. naphthophila*	20	6.5	75	25.4	[86]
*T. naphthophila* F226G	20	6.5	65	33.0	[86]
*T. naphthophila* F226G/F414S	20	6.5	60	61.1	[86]
*P. tritici SWRI145*	30	7.5	50	44.8	[87]
*L. bulgaricus* L3	16	6.2	45	44.7	[88]

**Table 2:** Statistics of immobilizer used in Bgal and its catalytic ability improvement.

Immobilizer	Enzyme Source	Catalytic Ability Improvement (Compared with Free Enzymes)				Method	References
		Activity	Stability	pH	Temperature		
Halloysite	*B. bifidum*	-	√	√	√	Physical adsorption	[85]
Agar-agar gel matrix	*Enterobacter aerogenes*	-	√	-	√	Entrapment	[103]
Magnetic nanoparticles	*Thermotoga maritima*	√	√	-	-	Physical adsorption	[67]
Iron oxide super-paramagnetic beads	*A. oryzae*	-	√	-	-	Physical adsorption	[75]
Anion-exchange resin	*A. oryzae*	√	√	-	√	Covalent binding and cross-linking	[101]
Polymer sodium alginate	*A. oryzae*	-	√	√	-	Physical adsorption	[104]
Hydrogel microparticles	*A. oryzae*	√	√	-	-	Affinity binding	[105]
Electrospun gelatin nanofiber mats	*A. oryzae*	√	√	-	√	Affinity binding	[106]
Gelatin nanofibers	*A. oryzae*	√	√	-	√	Entrapment	[106]
UV-cured epoxy-based polymeric film	*E. coli*	√	√	-	-	Affinity binding	[81]
Magnetic cellulose-based supports	*K. lactis*	√	√	-	-	Affinity binding	[107]
Genipin-activated chitosan	*K. lactis*	-	√	-	-	Physical adsorption	[108]
Genipin to cross-link *K. lactis*	*K. lactis*	-	√	-	√	Cross-link	[102]

“√”, Mentioned. “-”, Not mentioned.

**Table 3:** Surface display in the application of Bgal-catalyzed synthesis of GOS.

Source of Enzyme	Support Material	Display Vector	Temperature (°C)	Reaction Batch	Max Yield (%)	References
*Flavobacterium alginum*	-	*E. coli*	20	6	31.63	[113]
*A. oryzae*	-	*Y. lipolytica*	60	10	51	[114]
*A. oryzae*	Cotton fiber	*K. phaffii*	40	23	50.3	[115]
*A. oryzae*	Cotton fiber	*K. phaffii*	40	16	32.63	[116]
*L. delbrueckii* subsp. *bulgaricus*	-	*L. plantarum*	30	5	32	[117]
*L. reuteri*	-	*L. plantarum*	-	-	-	[117]

## Data Availability

No new data were created or analyzed in this study.

## References

[1] Arnold J.W., Roach J., Fabela S., Moorfield E., Ding S., Blue E., Dagher S., Magness S., Tamayo R., Bruno-Barcena J.M. (2021). The pleiotropic effects of prebiotic galacto-oligosaccharides on the aging gut. Microbiome.

[2] Yadav M.K., Kumari I., Singh B., Sharma K.K., Tiwari S.K. (2022). Probiotics, prebiotics and synbiotics: Safe options for next-generation therapeutics. Appl. Microbiol. Biotechnol..

[3] Sergeev I.N., Aljutaily T., Walton G., Huarte E. (2020). Effects of synbiotic supplement on human gut microbiota, body composition and weight loss in obesity. Nutrients.

[4] Massot-Cladera M., Rigo-Adrover M.d.M., Herrero L., Franch À., Castell M., Vulevic J., Pérez-Cano F.J., Lagunas M.J.R. (2022). A Galactooligosaccharide product decreases the rotavirus infection in suckling rats. Cells.

[5] Thompson R.S., Bowers S.J., Vargas F., Hopkins S., Kelley T., Gonzalez A., Lowry C.A., Dorrestein P.C., Vitaterna M.H., Turek F.W. (2024). A prebiotic diet containing galactooligosaccharides and polydextrose produces dynamic and reproducible changes in the gut microbial ecosystem in male rats. Nutrients.

[6] Wang K., Duan F., Sun T., Zhang Y., Lu L. (2024). Galactooligosaccharides: Synthesis, metabolism, bioactivities and food applications. Crit. Rev. Food Sci. Nutr..

[7] Yazawa K., Imai K., Tamura Z. (1978). Oligosaccharides and polysaccharides specifically utilizable by Bifidobacteria. Chem. Pharm. Bull..

[8] Wiciński M., Sawicka E., Gębalski J., Kubiak K., Malinowski B. (2020). Human milk oligosaccharides: Health benefits, potential applications in infant formulas, and pharmacology. Nutrients.

[9] Maráz A., Kovács Z., Benjamins E., Pázmándi M. (2022). Recent developments in microbial production of high-purity galacto-oligosaccharides. World J. Microbiol. Biotechnol..

[10] Vera C., Córdova A., Aburto C., Guerrero C., Suárez S., Illanes A. (2016). Synthesis and purification of galacto-oligosaccharides: State of the art. World J. Microbiol. Biotechnol..

[11] Angima G., Qu Y., Park S.H., Dallas D.C. (2024). Prebiotic strategies to manage lactose intolerance symptoms. Nutrients.

[12] Ye L., Jiang Y., Zhang M. (2022). Crosstalk between glucose metabolism, lactate production and immune response modulation. Cytokine Growth Factor Rev..

[13] Dou Y., Yu X., Luo Y., Chen B., Ma D., Zhu J. (2022). Effect of fructooligosaccharides supplementation on the gut microbiota in human: A systematic review and meta-analysis. Nutrients.

[14] Mei Z., Yuan J., Li D. (2022). Biological activity of galacto-oligosaccharides: A review. Front. Microbiol..

[15] Muñoz-Labrador A., Kolida S., Rastall R.A., Methven L., Lebrón-Aguilar R., Quintanilla-López J.E., Galindo-Iranzo P., Javier Moreno F., Hernandez-Hernandez O. (2024). Prebiotic potential of new sweeteners based on the simultaneous biosynthesis of galactooligosaccharides and enzymatically modified steviol glycosides. Food Chem..

[16] Juers D.H., Matthews B.W., Huber R.E. (2012). *LacZ* β-galactosidase: Structure and function of an enzyme of historical and molecular biological importance. Protein Sci..

[17] Catanzaro R., Sciuto M., Marotta F. (2021). Lactose intolerance: An update on its pathogenesis, diagnosis, and treatment. Nutr. Res..

[18] Hsu C.A., Lee S.L., Chou C.C. (2007). Enzymatic production of galactooligosaccharides by β-galactosidase from *Bifidobacterium longum* BCRC 15708. J. Agric. Food Chem..

[19] Sabater C., Fara A., Palacios J., Corzo N., Requena T., Montilla A., Zárate G. (2019). Synthesis of prebiotic galactooligosaccharides from lactose and lactulose by dairy propionibacteria. Food Microbiol..

[20] Oniszczuk A., Oniszczuk T., Gancarz M., Szymańska J. (2021). Role of gut microbiota, probiotics and prebiotics in the cardiovascular diseases. Molecules.

[21] Martínez-González A.E., Andreo-Martínez P. (2020). Prebioticos, probioticos y trasplante de microbiota fecal en el autismo: Una revision sistematica. Rev. Psiquiatr. Salud Ment..

[22] Zhao J., Cao Y., Miao Y., Guo H., Wang Z., Liang X., Guan X., Sun R., Zhang X., Nie C. (2025). Structure, enzymatic production, biological activities, and food applications of galacto-oligosaccharides: A Review. J. Nutr..

[23] Weijers C.A.G.M., Franssen M.C.R., Visser G.M. (2008). Glycosyltransferase-catalyzed synthesis of bioactive oligosaccharides. Biotechnol. Adv..

[24] Ambrogi V., Bottacini F., Mac Sharry J., van Breen J., O’Keeffe E., Walsh D., Schoemaker B., Cao L., Kuipers B., Lindner C. (2021). Bifidobacterial β-galactosidase-mediated production of galacto-oligosaccharides: Structural and preliminary functional assessments. Front. Microbiol..

[25] Souza A.F.C.e., Gabardo S., Coelho R.d.J.S. (2022). Galactooligosaccharides: Physiological benefits, production strategies, and industrial application. J. Biotechnol..

[26] Movahedpour A., Ahmadi N., Ghalamfarsa F., Ghesmati Z., Khalifeh M., Maleksabet A., Shabaninejad Z., Taheri-Anganeh M., Savardashtaki A. (2022). β-galactosidase: From its source and applications to its recombinant form. Biotechnol. Appl. Biochem..

[27] Yan Y., Guan W., Li X., Gao K., Xu X., Liu B., Zhang W., Zhang Y. (2021). β-galactosidase GALA from *Bacillus circulans* with high transgalactosylation activity. Bioengineered.

[28] Sharma S.K., Poudel Sharma S., Leblanc R.M. (2021). Methods of detection of β-galactosidase enzyme in living cells. Enzym. Microb. Technol..

[29] Lu L., Guo L., Wang K., Liu Y., Xiao M. (2020). β-Galactosidases: A great tool for synthesizing galactose-containing carbohydrates. Biotechnol. Adv..

[30] Ureta M.M., Martins G.N., Figueira O., Pires P.F., Castilho P.C., Gomez-Zavaglia A. (2021). Recent advances in β-galactosidase and fructosyltransferase immobilization technology. Crit. Rev. Food Sci. Nutr..

[31] Beckwith J.R. (1967). Regulation of the lac operon: Recent studies on the regulation of lactose metabolism in *Escherichia coli* support the operon model. Science.

[32] Ruiz-Ramírez S., Jiménez-Flores R. (2023). Invited review: Properties of β-galactosidases derived from *Lactobacillaceae* species and their capacity for galacto-oligosaccharide production. J. Dairy Sci..

[33] Zhou Y., Liu Y., Gao F., Xia Z., Zhang Z., Addai F.P., Zhu Y., Chen J., Lin F., Chen D. (2024). β-galactosidase: Insights into source variability, genetic engineering, immobilisation and diverse applications in food, industry and medicine. Int. J. Dairy Technol..

[34] Kalathinathan P., Sain A., Pulicherla K., Kodiveri Muthukaliannan G. (2023). A review on the various sources of β-galactosidase and its lactose hydrolysis property. Curr. Microbiol..

[35] Liu P., Chen Y., Ma C., Ouyang J., Zheng Z. (2025). β-galactosidase: A traditional enzyme given multiple roles through protein engineering. Crit. Rev. Food Sci. Nutr..

[36] Hu Y., Shen Y., Song Y., Yang Y., Shan Y., Zhang R., Zhao J. (2025). β-galactanase: An effective tool for the degradation of plant β-D-galactan. World J. Microbiol. Biotechnol..

[37] Coelho A.I., Berry G.T., Rubio-Gozalbo M.E. (2015). Galactose metabolism and health. Curr. Opin. Clin. Nutr. Metab. Care.

[38] Cardelle-Cobas A., Villamiel M., Olano A., Corzo N. (2008). Study of galacto-oligosaccharide formation from lactose using pectinex ultra SP-L. J. Sci. Food Agric..

[39] Zhang M., Luo L., Liu S., Hu H., Huang R., Sun Y., Lei H., Wei X. (2021). Detection of galactooligosaccharides with high lactose interference in infant formula using a simple single epimer chromatography. Food Chem..

[40] Slavin J. (2013). Fiber and prebiotics: Mechanisms and health benefits. Nutrients.

[41] Torres D.P.M., Gonçalves M.d.P.F., Teixeira J.A., Rodrigues L.R. (2010). Galacto-oligosaccharides: Production, properties, applications, and significance as prebiotics. Compr. Rev. Food Sci. Food Saf..

[42] Singh P., Arora S., Rao P.S., Kathuria D., Sharma V., Singh A.K. (2022). Effect of process parameters on the β-galactosidase hydrolysis of lactose and galactooligosaccharide formation in concentrated skim milk. Food Chem..

[43] Gitzelmann R. (1995). Galactose-1-phosphate in the pathophysiology of galactosemia. Eur. J. Pediatr..

[44] Lorántfy B., Johanson A., Faria-Oliveira F., Franzén C.J., Mapelli V., Olsson L. (2019). Presence of galactose in precultures induces *lacS* and leads to short lag phase in lactose-grown *Lactococcus lactis* cultures. J. Ind. Microbiol. Biotechnol..

[45] Zhao J., Wang Z., Jin Q., Feng D., Lee J. (2023). Isomerization of galactose to tagatose: Recent advances in non-enzymatic isomerization. J. Agric. Food Chem..

[46] Kong S., Huang X., Cao H., Bai Y., Che Q., Nie H., Su Z. (2022). Anti-obesity effects of galacto-oligosaccharides in obese rats. Eur. J. Pharmacol..

[47] Mistry R.H., Liu F., Borewicz K., Lohuis M.A.M., Smidt H., Verkade H.J., Tietge U.J.F. (2020). Long-term β-galacto-oligosaccharides supplementation decreases the development of obesity and insulin resistance in mice fed a western-type diet. Mol. Nutr. Food Res..

[48] Hoang N.T., Tóth K., Stacey G. (2020). The role of microRNAs in the legume–*Rhizobium* nitrogen-fixing symbiosis. J. Exp. Bot..

[49] Tang J., Xu L., Zeng Y., Gong F. (2021). Effect of gut microbiota on LPS-induced acute lung injury by regulating the TLR4/NF-kB signaling pathway. Int. Immunopharmacol..

[50] Sun C., Hao B., Pang D., Li Q., Li E., Yang Q., Zou Y., Liao S., Liu F. (2022). Diverse galactooligosaccharides differentially reduce LPS-induced inflammation in macrophages. Foods.

[51] Wang G., Wang H., Jin Y., Xiao Z., Umar Yaqoob M., Lin Y., Chen H., Wang M. (2022). Galactooligosaccharides as a protective agent for intestinal barrier and its regulatory functions for intestinal microbiota. Food Res. Int..

[52] Wang G., Sun W., Pei X., Jin Y., Wang H., Tao W., Xiao Z., Liu L., Wang M. (2021). Galactooligosaccharide pretreatment alleviates damage of the intestinal barrier and inflammatory responses in LPS-challenged mice. Food Funct..

[53] Wu Y., Zhang X., Liu X., Zhao Z., Tao S., Xu Q., Zhao J., Dai Z., Zhang G., Han D. (2024). Galactooligosaccharides and *Limosilactobacillus reuteri* synergistically alleviate gut inflammation and barrier dysfunction by enriching *Bacteroides acidifaciens* for pentadecanoic acid biosynthesis. Nat. Commun..

[54] Roselli M., Maruszak A., Grimaldi R., Harthoorn L., Finamore A. (2022). Galactooligosaccharide treatment alleviates DSS-induced colonic inflammation in Caco-2 cell model. Front. Nutr..

[55] Fischer C., Kleinschmidt T. (2018). Synthesis of galactooligosaccharides in milk and whey: A review. Compr. Rev. Food Sci. Food Saf..

[56] Urrutia P., Mateo C., Guisan J.M., Wilson L., Illanes A. (2013). Immobilization of *Bacillus circulans* β-galactosidase and its application in the synthesis of galacto-oligosaccharides under repeated-batch operation. Biochem. Eng. J..

[57] Erich S., Kuschel B., Schwarz T., Ewert J., Böhmer N., Niehaus F., Eck J., Lutz-Wahl S., Stressler T., Fischer L. (2015). Novel high-performance metagenome β-galactosidases for lactose hydrolysis in the dairy industry. J. Biotechnol..

[58] de Albuquerque T.L., de Sousa M., Gomes e Silva N.C., Girão Neto C.A.C., Gonçalves L.R.B., Fernandez-Lafuente R., Rocha M.V.P. (2021). β-galactosidase from *Kluyveromyces lactis*: Characterization, production, immobilization and applications—A review. Int. J. Biol. Macromol..

[59] Julio-Gonzalez L.C., Hernández-Hernández O., Javier Moreno F., Jimeno M.L., Doyagüez E.G., Olano A., Corzo N. (2020). Hydrolysis and transgalactosylation catalysed by β-galactosidase from brush border membrane vesicles isolated from pig small intestine: A study using lactulose and its mixtures with lactose or galactose as substrates. Food Res. Int..

[60] Maity M., Majumdar S., Bhattacharyya D.K., Bhowal J., Das A., Barui A. (2023). Evaluation of prebiotic properties of galactooligosaccharides produced by transgalactosylation using partially purified β-galactosidase from *Enterobacter aerogenes* KCTC2190. Appl. Biochem. Biotechnol..

[61] Kolesovs S., Semjonovs P. (2023). Microalgal conversion of whey and lactose containing substrates: Current state and challenges. Biodegradation.

[62] Zhao J.C., Mu Y.L., Gu X.Y., Xu X.N., Guo T.T., Kong J. (2022). Site-directed mutation of β-galactosidase from *Streptococcus thermophilus* for galactooligosaccharide-enriched yogurt making. J. Dairy Sci..

[63] Katoch G.K., Nain N., Kaur S., Rasane P. (2022). Lactose intolerance and its dietary management: An update. J. Am. Nutr. Assoc..

[64] Zhang X., Yao C., Wang T., Zhao H., Zhang B. (2021). Production of high-purity galacto-oligosaccharides (GOS) by *Lactobacillus*-derived β-galactosidase. Eur. Food Res. Technol..

[65] Zerva A., Limnaios A., Kritikou A.S., Thomaidis N.S., Taoukis P., Topakas E. (2021). A novel thermophile β-galactosidase from *Thermothielavioides terrestris* producing galactooligosaccharides from acid whey. New Biotechnol..

[66] Saburi W., Ota T., Kato K., Tagami T., Yamashita K., Yao M., Mori H. (2023). Function and structure of *Lacticaseibacillus casei* GH35 β-galactosidase LBCZ_0230 with high hydrolytic activity to lacto-N-biose I and galacto-N-biose. J. Appl. Glycosci..

[67] Alnadari F., Xue Y., Almakas A., Mohedein A., Samie A., Abdel-Shafi M., Abdin M. (2021). Large batch production of galactooligosaccharides using β-glucosidase immobilized on chitosan-functionalized magnetic nanoparticle. J. Food Biochem..

[68] Frenzel M., Zerge K., Clawin-Rädecker I., Lorenzen P. (2014). Comparison of the galacto-oligosaccharide forming activity of different β-galactosidases. LWT Food Sci. Technol..

[69] Serey M., Vera C., Guerrero C., Illanes A. (2021). Immobilization of *Aspergillus oryzae* β-galactosidase in cation functionalized agarose matrix and its application in the synthesis of lactulose. Int. J. Biol. Macromol..

[70] Pan C., Hu B., Li W., Sun Y., Ye H., Zeng X. (2009). Novel and efficient method for immobilization and stabilization of β-d-galactosidase by covalent attachment onto magnetic Fe_3_O_4_–chitosan nanoparticles. J. Mol. Catal. B Enzym..

[71] Rodriguez-Colinas B., Fernandez-Arrojo L., Ballesteros A.O., Plou F.J. (2014). Galactooligosaccharides formation during enzymatic hydrolysis of lactose: Towards a prebiotic-enriched milk. Food Chem..

[72] Goulas T.K., Goulas A.K., Tzortzis G., Gibson G.R. (2007). Molecular cloning and comparative analysis of four β-galactosidase genes from *Bifidobacterium bifidum* NCIMB41171. Appl. Microbiol. Biotechnol..

[73] Wu Y., Yuan S., Chen S., Wu D., Chen J., Wu J. (2013). Enhancing the production of galacto-oligosaccharides by mutagenesis of *Sulfolobus solfataricus* β-galactosidase. Food Chem..

[74] Huerta M., San Martín A., Arancibia B., Cornejo F.A., Arenas F., Illanes A., Guerrero C., Vera C. (2024). Integrating the enzymatic syntheses of lactulose, epilactose and galacto-oligosaccharides. Food Bioprod. Process..

[75] Córdova A., Aburto C., Carrasco V., Guerrero C., Henriquez P., Astudillo-Castro C., Catalán S., Poerio T., Mazzei R. (2024). Development of a magnetic responsive biocatalalytic membrane reactor (MR-BMR) to produce galacto-oligosaccharides (GOS) using saturated lactose concentrations. Process Saf. Environ. Prot..

[76] Füreder V., Rodriguez-Colinas B., Cervantes F.V., Fernandez-Arrojo L., Poveda A., Jimenez-Barbero J., Ballesteros A.O., Plou F.J. (2020). Selective synthesis of galactooligosaccharides containing β(1→3) linkages with β-galactosidase from *Bifidobacterium bifidum* (Saphera). J. Agric. Food Chem..

[77] Miao M., Li S., Yang S., Yan Q., Xiang Z., Jiang Z. (2024). In situ galacto-oligosaccharides synthesis in whey powder fortified milk by a modified β-galactosidase and its effect on the techno-functional characteristics of yogurt. J. Agric. Food Chem..

[78] Zhao J., Niu D., Jin Z., Liu J., Ni D., McHunu N.P., Fan R., Singh S., Wang Z. (2025). Engineered β-galactosidase catalyzes lactose to prebiotics in situ in raw milk. Biotechnol. Lett..

[79] Noriega M.A., Rico-Rodríguez F., Rosales J.D., Serrato-Bermúdez J.C. (2024). Kinetics of galactooligosaccharides (GOS) production with two β-galactosidases: Metallic ion effect and mathematical model. Chem. Eng. Res. Des..

[80] Limnaios A., Tsevdou M., Zafeiri E., Topakas E., Taoukis P. (2024). Cheese and yogurt by-products as valuable ingredients for the production of prebiotic oligosaccharides. Dairy.

[81] Beyler-Çigil A., Danis O., Sarsar O., Kahraman M.V., Ogan A., Demir S. (2021). Optimizing the immobilization conditions of β-galactosidase on UV-cured epoxy-based polymeric film using response surface methodology. J. Food Biochem..

[82] Wang G., Jiang J., Liu L., Huang J. (2024). Recombinant beta-galactosidase derived from *Enterobacter cloacae* Zjut HJ2001 for efficient biotransformation of galactooligosaccharides. Biochem. Eng. J..

[83] Arsov A., Ivanov I., Tsigoriyna L., Petrov K., Petrova P. (2022). In vitro production of galactooligosaccharides by a novel β-galactosidase of *Lactobacillus bulgaricus*. Int. J. Mol. Sci..

[84] Fara A., Sabater C., Palacios J., Requena T., Montilla A., Zárate G. (2020). Prebiotic galactooligosaccharides production from lactose and lactulose by *Lactobacillus delbrueckii* subsp. *bulgaricus* CRL450. Food Funct..

[85] Tizchang S., Khiabani M.S., Mokarram R.R., Hamishehkar H., Mohammadi N.S., Chisti Y. (2021). Immobilization of β-galactosidase by halloysite-adsorption and entrapment in a cellulose nanocrystals matrix. Biochim. Biophys. Acta (BBA) Gen. Subj..

[86] Lyu J., Zhang J., Zhu J., Chen S., Han T., Zhang Y., Gao R., Xie G., Guo Z. (2022). Molecular dynamics simulation guided distal mutation of *Thermotoga naphthophila* β-glucosidase for significantly enhanced synthesis of galactooligosaccharides and expanded product scope. Int. J. Biol. Macromol..

[87] Jin X., Cheng Z., Zhang Y., Petrova P., Petrov K., Zhang W., Mu W. (2025). A new β-galactosidase from *Pseudomonas tritici* SWRI145 for efficient bioproduction of galactooligosaccharides. Foods.

[88] Duan F., Zhao R., Yang J., Xiao M., Lu L. (2021). Integrated utilization of dairy whey in probiotic β-galactosidase production and enzymatic synthesis of galacto-oligosaccharides. Catalysts.

[89] Larkin M.A., Blackshields G., Brown N.P., Chenna R., McGettigan P.A., McWilliam H., Valentin F., Wallace I.M., Wilm A., Lopez R. (2007). Clustal W and Clustal X version 2.0. Bioinformatics.

[90] Boutet E., Lieberherr D., Tognolli M., Schneider M., Bairoch A., Edwards D. (2007). UniProtKB/Swiss-Prot. Plant Bioinformatics: Methods and Protocols.

[91] David A., Islam S., Tankhilevich E., Sternberg M.J.E. (2022). The AlphaFold database of protein structures: A biologist’s guide. J. Mol. Biol..

[92] Wilkins M.R., Gasteiger E., Bairoch A., Sanchez J.C., Williams K.L., Appel R.D., Hochstrasser D.F., Link A.J. (1999). Protein identification and analysis tools in the ExPASy server. 2-D Proteome Analysis Protocols.

[93] Liao L., Han L., Sun Y., Du X., Wu Y., Luo J., Li J., Du G., Zhang G. (2026). Engineering β-galactosidase with enhanced catalytic and transglycosylation activity for GOS production. J. Agric. Food Chem..

[94] Xiang Z., Miao M., Jiang Z., Yan Q., Yang S. (2025). Efficient mutagenesis strategy based on nonpolar amino acids scanning for the improvement of transglycosylation ability of β-galactosidases. J. Agric. Food Chem..

[95] Yu H., Wang Y., Yang Z., Ying J., Guan F., Liu B., Miao M., Mohamed A., Wei X., Yang Y. (2025). Enhancing the synthesis efficiency of galacto-oligosaccharides of a β-galactosidase from *Paenibacillus barengoltzii* by engineering the active and distal sites. Food Chem..

[96] Sayers E.W., Beck J., Bolton E.E., Bourexis D., Brister J.R., Canese K., Comeau D.C., Funk K., Kim S., Klimke W. (2020). Database resources of the national center for biotechnology information. Nucleic Acids Res..

[97] Duan F., Sun T., Zhang J., Wang K., Wen Y., Lu L. (2022). Recent innovations in immobilization of β-galactosidases for industrial and therapeutic applications. Biotechnol. Adv..

[98] Khalid N., Kalsoom U., Ahsan Z., Bilal M. (2022). Non-magnetic and magnetically responsive support materials immobilized peroxidases for biocatalytic degradation of emerging dye pollutants—A review. Int. J. Biol. Macromol..

[99] Tacias-Pascacio V.G., Morellon-Sterling R., Castañeda-Valbuena D., Berenguer-Murcia Á., Kamli M.R., Tavano O., Fernandez-Lafuente R. (2021). Immobilization of papain: A review. Int. J. Biol. Macromol..

[100] Tang S.C.N., Lo I.M.C. (2013). Magnetic nanoparticles: Essential factors for sustainable environmental applications. Water Res..

[101] Antošová M., Adamíková J.K., Polakovič M. (2025). Galactooligosaccharide production using immobilized *Aspergillus oryzae* β-galactosidase, Part I: Characterization and influence of reaction conditions. Int. J. Mol. Sci..

[102] Xuan Z., Wang K., Duan F., Lu L. (2024). Non-carrier immobilization of yeast cells by genipin crosslinking for the synthesis of prebiotic galactooligosaccharides from plant-derived galactose. Int. J. Biol. Macromol..

[103] Maity M., Bhattacharyya A., Bhowal J. (2021). Production and immobilization of β-galactosidase isolated from *Enterobacter aerogenes* KCTC2190 by entrapment method using agar-agar organic matrix. Appl. Biochem. Biotechnol..

[104] Souza C.J.F., Garcia-Rojas E.E., Souza C.S.F., Vriesmann L.C., Vicente J., de Carvalho M.G., Petkowicz C.L.O., Favaro-Trindade C.S. (2019). Immobilization of β-galactosidase by complexation: Effect of interaction on the properties of the enzyme. Int. J. Biol. Macromol..

[105] Suvarli N., Wenger L., Serra C., Perner-Nochta I., Hubbuch J., Wörner M. (2022). Immobilization of β-galactosidase by encapsulation of enzyme-conjugated polymer nanoparticles inside hydrogel microparticles. Front. Bioeng. Biotechnol..

[106] Sass A.C., Jördening H.J. (2020). Immobilization of β-galactosidase from *Aspergillus oryzae* on electrospun gelatin nanofiber mats for the production of galactooligosaccharides. Appl. Biochem. Biotechnol..

[107] Gennari A., Simon R., Sperotto N.D.d.M., Bizarro C.V., Basso L.A., Machado P., Benvenutti E.V., Da Cas Viegas A., Nicolodi S., Renard G. (2022). One-step purification of a recombinant beta-galactosidase using magnetic cellulose as a support: Rapid immobilization and high thermal stability. Bioresour. Technol..

[108] Lima P.C., Gazoni I., de Carvalho A.M.G., Bresolin D., Cavalheiro D., de Oliveira D., Rigo E. (2021). β-galactosidase from *Kluyveromyces lactis* in genipin-activated chitosan: An investigation on immobilization, stability, and application in diluted UHT milk. Food Chem..

[109] Prabhakar T., Giaretta J., Zulli R., Rath R.J., Farajikhah S., Talebian S., Dehghani F. (2025). Covalent immobilization: A review from an enzyme perspective. Chem. Eng. J..

[110] Teymennet-Ramírez K.V., Martínez-Morales F., Trejo-Hernández M.R. (2022). Yeast surface display system: Strategies for improvement and biotechnological applications. Front. Bioeng. Biotechnol..

[111] van Bloois E., Winter R.T., Kolmar H., Fraaije M.W. (2011). Decorating microbes: Surface display of proteins on *Escherichia coli*. Trends Biotechnol..

[112] Park M. (2020). Surface display technology for biosensor applications: A review. Sensors.

[113] Gao K., Yu K., Sun J., Mao X., Dong H. (2025). Engineering an *Escherichia coli* surface display platform based on an autotransporter from *Stenotrophomonas maltophilia*: Autodisplay of enzymes with low to high molecular weight. J. Biotechnol..

[114] An J., Zhang L., Li L., Liu D., Cheng H., Wang H., Nawaz M.Z., Cheng H., Deng Z. (2016). An alternative approach to synthesizing galactooligosaccharides by cell-surface display of β-galactosidase on *Yarrowia lipolytica*. J. Agric. Food Chem..

[115] Chen T., Wang S., Niu H., Yang G., Wang S., Wang Y., Zhou C., Yu B., Yang P., Sun W. (2023). Biofilm-based biocatalysis for galactooligosaccharides production by the surface display of β-galactosidase in *Pichia pastoris*. Int. J. Mol. Sci..

[116] Zhou C., Zhu Y., Ren P., Leng J., Xia X., Chen T., Sun W., Yang P., Niu H., Chen Y. (2025). Construction of an efficient enzyme-cell@material biocatalyst through the biofilm immobilization of *Komagataella phaffii* for continuous biocatalysis. Bioresour. Technol..

[117] Pham M.L., Tran A.M., Kittibunchakul S., Nguyen T.T., Mathiesen G., Nguyen T.H. (2019). Immobilization of β-galactosidases on the *Lactobacillus* cell surface using the peptidoglycan-binding motif LysM. Catalysts.

[118] Wang F., Liu X., Song T., Pei C., Huang Q., Jiang H., Xi H. (2023). First display of haloalkane dehalogenase LinB on the surface of *Bacillus subtilis* spore. Protein Pept. Lett..

[119] Li Z., Chen Y., Liu D., Zhao N., Cheng H., Ren H., Guo T., Niu H., Zhuang W., Wu J. (2015). Involvement of glycolysis/gluconeogenesis and signaling regulatory pathways in *Saccharomyces cerevisiae* biofilms during fermentation. Front. Microbiol..

[120] Li Y., Wang X., Zhou N.Y., Ding J. (2024). Yeast surface display technology: Mechanisms, applications, and perspectives. Biotechnol. Adv..

[121] Shi L., Zhang J., Zhao M., Tang S., Cheng X., Zhang W., Li W., Liu X., Peng H., Wang Q. (2021). Effects of polyethylene glycol on the surface of nanoparticles for targeted drug delivery. Nanoscale.

[122] Ponnusamy V., Sankaranarayanan M. (2023). Targeted gene manipulation of leloir pathway genes for the constitutive expression of β-galactosidase and its transgalactosylation product galacto-oligosaccharides from *Kluyveromyces lactis* GG799 and knockout strains. Enzym. Microb. Technol..

[123] Gupta D., Bhattacharjee O., Mandal D., Sen M.K., Dey D., Dasgupta A., Kazi T.A., Gupta R., Sinharoy S., Acharya K. (2019). CRISPR-Cas9 system: A new-fangled dawn in gene editing. Life Sci..

[124] Zhao J., Mu T., Niu D., Ding Z., McHunu N.P., Zhang M., Singh S., Wang Z. (2025). Metabolic engineering of *Bacillus licheniformis* for high-yield L-lactic acid and galactooligosaccharide retention in complementary synbiotics production. Microorganisms.

[125] Nguyen T.T., Nguyen H.M., Geiger B., Mathiesen G., Eijsink V.G.H., Peterbauer C.K., Haltrich D., Nguyen T.H. (2015). Heterologous expression of a recombinant lactobacillal β-galactosidase in *Lactobacillus plantarum*: Effect of different parameters on the sakacin P-based expression system. Microb. Cell Fact..

[126] Wei W., Qi D., Zhao H.Z., Lu Z.Z., Lv F., Bie X. (2013). Synthesis and characterisation of galactosyl glycerol by β-galactosidase catalysed reverse hydrolysis of galactose and glycerol. Food Chem..

[127] Bartesaghi A., Merk A., Banerjee S., Matthies D., Wu X., Milne J.L.S., Subramaniam S. (2015). 2.2 Å resolution cryo-EM structure of β-galactosidase in complex with a cell-permeant inhibitor. Science.

[128] Sanità G., Maresca E., Capaldi S., Casillo A., Aulitto M., Donadio F., Pape T., Corsaro M.M., Esposito E., Contursi P. (2026). CryoEM structural analysis of a thermophilic galactooligosaccharides-producer β-galactosidase unravels an uncommon oligomeric structure. Int. J. Biol. Macromol..

